# Bilateral Hand Salvage With Simultaneous Pedicled Groin Flaps in an Immunocompromised Patient

**Published:** 2018-09-03

**Authors:** Sherise Epstein, Christopher M. Reid, Fernando Herrera, Reid A. Abrams, Ahmed S. Suliman

**Affiliations:** ^a^Division of Plastic Surgery, Department of Surgery, UC San Diego Medical Center, San Diego, Calif; ^b^Division of Plastic and Reconstructive Surgery, Medical University of South Carolina, Charleston; ^c^Department of Orthopedic Surgery, UC San Diego Medical Center, San Diego, Calif; ^d^Section of Plastic & Reconstructive Surgery, VA San Diego Healthcare System, San Diego, Calif

**Keywords:** bilateral hand salvage, hand reconstruction, groin flap, hand tumor, hand flap

**Video 1:** Preoperative photographs and plain films, remnants after resection, pedicled groin flaps, and postoperative function.

## DESCRIPTION

A 45-year-old immunosuppressed woman status post 3 prior kidney transplants presented with recurrent advanced squamous cell carcinoma (SCC) of the bilateral hands. She had multiple cardiac comorbidities, monocular blindness, and poor nutritional status. She desired maximal hand salvage and refused proximal amputation. A staged oncologic resection with reconstruction was planned.

## QUESTIONS

1. Describe the epidemiology of and risk factors for SCC of the hand.
2. Describe the presenting lesions and preoperative workup.
3. Describe the defects left after surgical excision and the goals for reconstruction.
4. Describe considerations affecting reconstructive options.

## DISCUSSION

The hands represent a mere 1% to 2% of total body surface area; yet, skin cancer of the hand accounts for 10% to 15% of all skin malignancies.^1^ Squamous cell carcinoma is the most common cutaneous malignancy of the hand.^1^^,^^2^ Compared with the general population, kidney transplant recipients have a significantly higher risk of SCC.^3^^,^^4^ This risk is particularly high among patients with Fitzpatrick skin types I-III.^3^ However, any site of chronic inflammation is at risk of developing SCC, of which some etiologies predominantly affect skin of color, often due to the social epidemiology of disease (eg, discoid lupus erythematosus, lupus vulgaris, arsenic exposure).^5^^,^^6^

This patient had large, fungating masses involving both dorsal and volar aspects of the index, middle, and ring fingers, second and third web spaces, and dorsum and palm of the bilateral hands. There was sparing of the thumb and the small finger of the right hand and the small finger of the left hand. There was a small superficial lesion over the distal left thumb that was not contiguous with larger lesions. Recent biopsy demonstrated moderately differentiated SCC. Squamous cell carcinomas of the web spaces and dorsal proximal phalanges have a high propensity for metastasis.^1^ This patient did not have palpable epitrochlear or axillary lymph nodes, and a positron emission tomography scan was negative. Switching from calcineurin inhibitors to sirolimus has been shown to have an antitumoral effect among kidney transplant recipients with SCC^7^; however, this patient's cardiac comorbidities precluded this potential adjuvant therapy.

The index, middle, and ring metacarpals were transected at the level of the proximal metadiaphyseal junction. There was little soft tissue available for wound coverage on the left and none on the right. The skin was taut, fibrous, and fragile due to prior local radiation therapy, long-term oral prednisone use, and malnourishment. The primary goal for reconstruction was maximizing sensation and function, so the patient could maintain her independence.

In general, skin grafts, local flaps, and free flaps are the preferred options for oncologic reconstructions, as distant pedicled flaps are not recommended because of the theoretical possibility of seeding a distant site with tumor cells.^8^ However, the extent of this patient's wound defects, tissue quality, anatomic structure, and medical comorbidities precluded these options. The initial reconstructive plan for the left hand was to utilize a fillet flap of the left index finger and either a distally based forearm flap or free tissue transfer on the right. However, after reexcision for margin control on the left hand, there was inadequate perfusion to the left index finger fillet flap. Furthermore, intraoperative evaluation revealed incomplete palmar arches bilaterally, precluding pedicled forearm flaps. Given the poor quality of her forearm vessels and incomplete collateral circulation, it was determined that free tissue transfer had an unacceptably low likelihood of success. The wounds were temporized with negative pressure wound therapy until clear margins were confirmed. Although unanticipated, we proceeded with simultaneous bilateral pedicled groin flaps after thorough discussions on morbidity. The patient's chronic immunosuppression and malnutrition significantly delayed wound healing, requiring twice as long as typical for peripheral wound ingrowth to allow for pedicle transection. However, pedicle transection and flap inset were successful and the donor sites healed well. After 3 months, she had good range of motion in the remaining digits with no pain. She was able to return to all preoperative activities and maintained independence.

Simultaneous bilateral pedicled groin flaps are feasible for bilateral hand salvage in patients with no local or regional reconstructive options and a low likelihood of success with free tissue transfer.

## Figures and Tables

**Figure 1:**
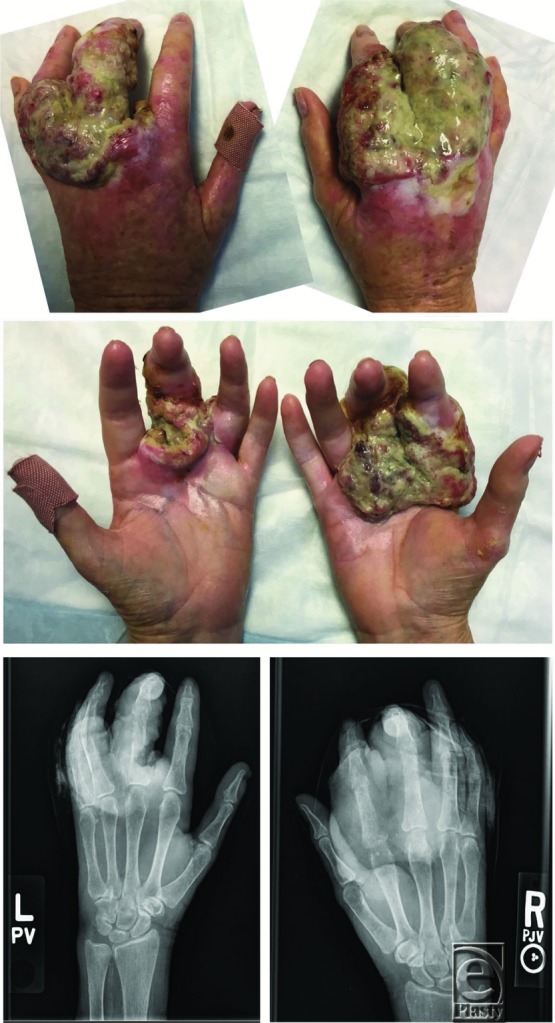
Preoperative photographs and plain films.

**Figure 2:**
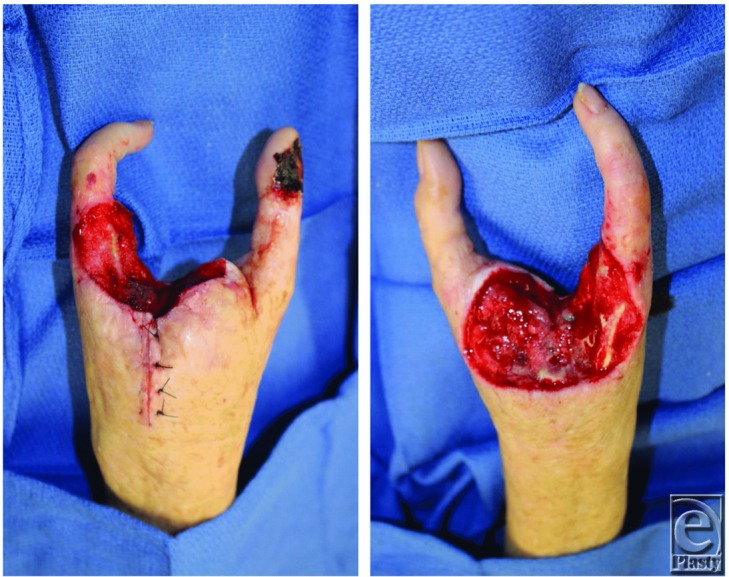
Remnants after resection.

**Figure 3:**
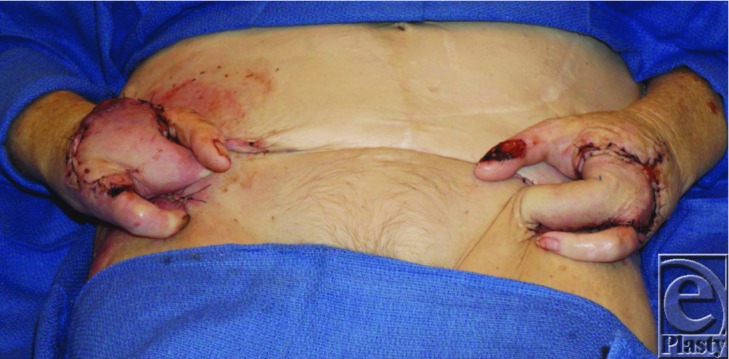
Pedicled groin flaps.

## References

[1] Maciburko SJ, Townley WA, Hollowood K, Giele HP (2012). Skin cancers of the hand: a series of 541 malignancies. Plast Reconstr Surg.

[2] Schiavon M, Mazzoleni F, Chiarelli A, Matano P (1988). Squamous cell carcinoma of the hand: fifty-five case reports. J Hand Surg Am.

[3] Gogia R, Binstock M, Hirose R, Boscardin WJ, Chren M-M, Arron ST (2013). Fitzpatrick skin phototype is an independent predictor of squamous cell carcinoma risk after solid organ transplantation. J Am Acad Dermatol.

[4] Comeau S, Jensen L, Cockfield SM, Sapijaszko M, Gourishankar S (2008). Non-melanoma skin cancer incidence and risk factors after kidney transplantation: a Canadian experience. Transplantation.

[5] Carter EE, Barr SG, Clarke AE (2016). The global burden of SLE: prevalence, health disparities and socioeconomic impact. Nat Rev Rheumatol.

[6] Thomas BP, Sasi K, Pallapati SC, Mathew A, Sreekanth R, Thomas M (2011). Malignant tumours of the hand and wrist. Indian J Plast Surg.

[7] Euvrard S, Morelon E, Rostaing L (2012). Sirolimus and secondary skin-cancer prevention in kidney transplantation. N Engl J Med.

[8] Haase SC, Chung KC, Wolfe SW, Hotchkiss RN, Pederson WC, Kozin SH, Cohen MS, Green DP Skin tumors. Green's Operative Hand Surgery.

